# Optically Reconfigurable Complementary Logic Gates Enabled by Bipolar Photoresponse in Gallium Selenide Memtransistor

**DOI:** 10.1002/advs.202205383

**Published:** 2023-04-19

**Authors:** Shania Rehman, Muhammad Asghar Khan, Honggyun Kim, Harshada Patil, Jamal Aziz, Kalyani D. Kadam, Malik Abdul Rehman, Muhammad Rabeel, Aize Hao, Karim Khan, Sungho Kim, Jonghwa Eom, Deok‐kee Kim, Muhammad Farooq Khan

**Affiliations:** ^1^ Department of Semiconductor System Engineering Sejong University Seoul 05006 Republic of Korea; ^2^ Department of Physics & Astronomy and Graphene Research Institute Sejong University Seoul 05006 Republic of Korea; ^3^ Department of Electrical Engineering Sejong University Seoul 05006 Republic of Korea; ^4^ Department of Convergence Engineering for Intelligent Drone Sejong University Seoul 05006 South Korea; ^5^ Department of Chemical Engineering New Uzbekistan University Tashkent 100007 Uzbekistan; ^6^ State Key Laboratory of Chemistry and Utilization of Carbon‐Based Energy Resources College of Chemistry Xinjiang University Urumqi Xinjiang 830017 P. R. China; ^7^ School of Mechanical Engineering Dongguan University of Technology Dongguan 523808 P. R. China

**Keywords:** charge trapping, gallium selenide, logic gates, memtransistors, resistive switching

## Abstract

To avoid the complexity of the circuit for in‐memory computing, simultaneous execution of multiple logic gates (OR, AND, NOR, and NAND) and memory behavior are demonstrated in a single device of oxygen plasma‐treated gallium selenide (GaSe) memtransistor. Resistive switching behavior with R_ON_/R_OFF_ ratio in the range of 10^4^ to 10^6^ is obtained depending on the channel length (150 to 1600 nm). Oxygen plasma treatment on GaSe film created shallow and deep‐level defect states, which exhibit carriers trapping/de‐trapping, that lead to negative and positive photoconductance at positive and negative gate voltages, respectively. This distinguishing feature of gate‐dependent transition of negative to positive photoconductance encourages the execution of four logic gates in the single memory device, which is elusive in conventional memtransistor. Additionally, it is feasible to reversibly switch between two logic gates by just adjusting the gate voltages, e.g., NAND/NOR and AND/NAND. All logic gates presented high stability. Additionally, memtransistor array (1×8) is fabricated and programmed into binary bits representing ASCII (American Standard Code for Information Interchange) code for the uppercase letter “N”. This facile device configuration can provide the functionality of both logic and memory devices for emerging neuromorphic computing.

## Graphical abstract

Resistive switching of oxygen plasma treated GaSe is showing a promising I_ON_/I_OFF_ ratio (10^4^–10^6^). Multiple highly stable logic gate operations are demonstrated in a single device based on gate and optical inputs. Most attractive feature of the devices is the re‐configurability that can switch between two logic gates by just adjusting the gate voltages, e.g., NAND/NOR and AND/NAND, OR/NOR, AND/NAND.

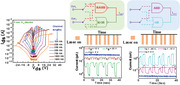

## 1 Introduction

With the emergence of the big data era, which refers to the massive combination of structured and complex unstructured data sets, it has become problematic to process this complex data by using conventional von Neumann computing, having separate processing and memory units.^[^ ^1^, ^2^, ^3^ ^]^ Neuromorphic computing, which imitates the human brain's ability to execute energy efficient processing of data sets, has gained considerable attention to fulfill these technical demands.^[^ ^4^, ^5^, ^6^, ^7^ ^]^ For example, even a single human brain neuron can perform a Boolean operation.^[^ ^8^ ^]^ Although the human brain inspired artificial neuromorphic system comprised of memory devices can process information efficiently, but typically it requires numerous memory devices to execute a single Boolean operation.^[^ ^9^, ^10^, ^11^, ^12^, ^13^, ^14^, ^15^ ^]^ For instance, four operation steps are required on four memory devices,^[^ ^16^ ^]^ or three third‐order elements ^[^ ^17^ ^]^ are necessary to perform Boolean operations.

Recently, 2D layered materials have attained considerable attention over metal oxides as potential candidates for their use in resistive memories and synaptic devices.^[^ ^18^, ^19^, ^20^, ^21^, ^22^ ^]^ These materials can perform proficiently due to inter‐layer van der Waals bonding and enhanced electrostatic control which may replace the silicon‐based memory components.^[^ ^23^, ^24^, ^25^ ^]^ Initially, the vertical geometry of memory devices has gained much interest due to low power consumption. However, the performance of vertical configuration is limited by the lack of control of the filament formation and consequently on the operating voltages. These findings overturned the concept of vertical device preference over lateral device structures and the research on lateral configurations increased. Hence, in 2015, resistive switching behavior was demonstrated in MoS_2_ with a lateral device structure.^[^ ^26^ ^]^ This lateral device with an additional gate electrode had shown enhanced device performance by providing extra functionalities. The SET voltage of the device was controlled by the gate electrode in a field effect transistor geometry. Additionally, this tuning of SET voltage and the conductance of the active layer by the gate electrode may eliminate the requirement for a selector device. Moreover, these three terminal structures can demonstrate complex functions such as hetero‐synaptic plasticity in neuromorphic computing which is difficult to imitate in a simple two‐terminal lateral/vertical configuration.^[^ ^26^, ^27^, ^28^ ^]^ Recently, Sangwan et al. ^[^ ^28^ ^]^ proposed the idea of memtransistor by combining a memristor with a transistor which is basically a multi‐terminal device having the characteristics of both memristor and transistor to demonstrate the advanced neuromorphic learning phenomena. Initially, synaptic devices use electrical stimulation only. Later, the progress of optogenetics in the field of neuroscience has motivated the incorporation of light signal into synaptic devices rather than including electrical signals only.^[^ ^29^, ^30^, ^31^ ^]^ So, a device with both optical and electrical input can have more application as compared to electrically stimulated device. Moreover, an interesting phenomenon of negative photoconductance (NPC) is also linked with light stimulation. Basically, when the semiconductors are illuminated with light having energy greater than the band gap of semiconductors, then excess electrons and holes are produced, which results in an increase of conductivity in semiconductors. This phenomenon is termed as positive photoconductance (PPC). Conversely, the decrease in conductivity on exposure to light is characterized as NPC. NPC has the potential to operate optoelectronic devices with low power intake and high‐speed frequency response.^[^ ^32^ ^]^ So, the combination of NPC and PPC in a single device is anticipated to increase the functionality of conventional optoelectronic devices.

In this report, the resistive switching characteristics of GaSe based lateral devices are explored. To initiate resistive switching and increase the current on/off ratio, we exposed our devices under oxygen plasma for different exposure times. Plasma induced trap states can hold the charges for long time duration due to their large capture and emission time constants. This feature is helpful to maintain the reliability of memory devices and provides good endurance and retention characteristics of the devices. The gate dependent transition of PPC to NPC is observed in GaSe memtranistor. This feature helps us develop multiple logic gates in a single device, inspired by the function of single neuron. This distinctive attribute of our device is usually unattainable in typical 2D materials‐based devices, as in MoS_2_ and ReS_2_ transistors, multiple transistors must be combined in series or parallel ^[^ ^33^, ^34^ ^]^ to perform a single Boolean operation. Here, in this report, four logic functions are implemented in single device.

## 2 Results and Discussion

**Figure 1:**
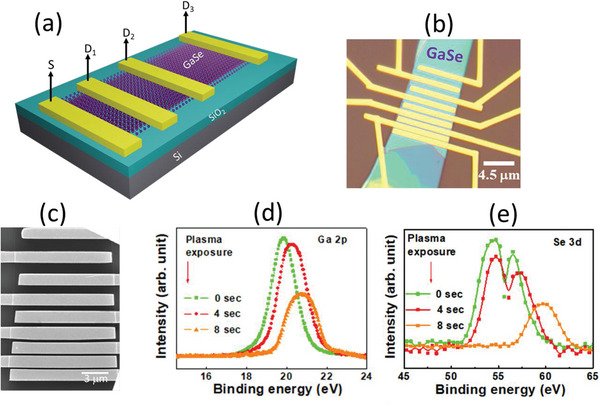
a) Schematic diagram of GaSe lateral transistor with one source “S” and various drains (D_1_, D_2_, and D_3_). b) Optical micrograph and c) SEM image of GaSe memtransistor. High resolution XPS spectra of (d) Ga 2p and e) Se 3d in pristine, 4 and 8 s oxygen plasma treated GaSe devices.

The schematic diagram, microscopic image, and FESEM image of our device are shown in **Figures** **1**a,b,c, respectively. The p‐type Si serves as a gate with 300 nm SiO_2_ as a gate dielectric whereas the GaSe is the active channel material for memtransistors. The source and drain of the GaSe memtransistor are formed by Cr/Au (6/40 nm). To confirm the thickness of GaSe we performed atomic force microscopy (AFM). The AFM image and height profile of the multilayer GaSe flake is shown in Figure S1 (Supporting Information). The thickness of the GaSe flake is estimated to be ≈42.1 nm according to the corresponding height profile presented in Figure S1(b) (Supporting Information).

Primarily, the structural and composition characteristic of GaSe and its oxide phases are investigated. Hence, Raman spectroscopy is utilized to study the vibrational modes of GaSe and its atop oxidation layer, see Figure S2 (Supporting Information) where we explore the Raman spectra of freshly exfoliated GaSe and oxygen‐plasma treated GaSe for 4 and 8 s. The spectrum of freshly exfoliated GaSe presented Raman modes of GaSe are observed at 134, 214, and 308 cm^−1^ which is consistent with previous reports.^[^ ^35^, ^36^ ^]^ The Raman modes are modified considerably when exposed to oxygen‐plasma for different time (4 and 8 s), therefore, the intensity of GaSe's Raman modes is decreased and few additional Raman modes emerged at frequencies ≈155, 161, 257 cm^−1^. Subsequently, after 8 s of oxygen‐plasma, the intensities of all GaSe related modes are dropped to ≈20% of their initial values. The degradation of these modes’ intensities shows that the crystal quality of the top GaSe layers is remarkably affected. The extrinsic additional modes are attributed to oxygen‐plasma treatment which encourage oxidation by‐products of GaSe (Ga_2_Se_3_, a‐Se, and *β*‐Ga_2_O_3_ phases).^[^ ^35^, ^36^ ^]^ The Raman modes at 155 and 257 cm^−1^ correspond to A_1_ mode of Ga_2_Se_3_ and amorphous selenium,^[^ ^36^ ^]^ respectively. As it can be seen in Figure S2 (Supporting Information), the intensity of oxidation related modes is considerable and detectable even after only 4 s of plasma treatment. After 8 s of oxygen plasma treatment, a Raman mode at 161 cm^−1^ emerges which is designated to Ga_2_O_3_.^[^ ^37^, ^38^ ^]^ The oxidation of GaSe occurs in the following steps, and it is expressed as ^[^ ^39^ ^]^

(1) $$GaSe+\;\frac{1}{4}O_{2}\;\to \;\frac{1}{3}Ga_{2}Se_{3}+\;\frac{1}{6}Ga_{2}O_{3}$$

(2) $$GaSe+\;\frac{3}{4}O_{2}\to \;\frac{1}{2}Ga_{2}O_{3}+3Se$$

(3) $$Ga_{2}Se_{3}+\;\frac{3}{2}O_{2}\to \;Ga_{2}O_{3\;}+3Se$$

By summarizing the whole discussion, we concluded that the top layers of the GaSe were oxidized, with different oxidation products (Ga_2_Se_3_, Ga_2_O_3_ and amorphous and crystalline selenium). Hence, the oxygen‐plasma treatment of GaSe leads to a defective structure that is comprised of GaSe layers capped with a combination of Ga_2_Se_3_, Ga_2_O_3_, and a‐Se.

To further verify the composition, X‐ray photoelectron spectroscopy (XPS) is performed as shown in Figure 1d,e where a comparative analysis has been made for Ga “2p3/2” XPS spectra of freshly cleaved GaSe and oxygen‐plasma treated GaSe for two different exposure times (4 and 8 s). Here, a broad peak is located at ≈19.8 eV for the freshly cleaved sample which is attributed to Ga “3d” peak of GaSe.^[^ ^36^, ^40^ ^]^ XPS peaks for the oxygen‐plasma treated samples for 4 and 8 sec become broadened where a significant change in the line shape and peak positions are observed. The broadening and shifting of peaks are associated with oxide formation.^[^ ^35^ ^]^ Figure 1d presents that the Se “3d” peak is observed at 54.7 eV ^[^ ^40^ ^]^ in the freshly exfoliated GaSe sample, which corresponds to the GaSe phase. After plasma treatment, the Se “3d” peaks are shifted and broadened. The broadening and shifting of the peak are indications of the formation of amorphous Se. After 8 s of oxygen plasma treatment, the peak is shifted to 60 eV, which is assigned to the formation of SeO.^[^ ^35^ ^]^

**Figure 2:**
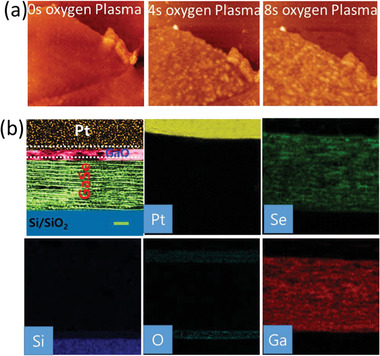
a) AFM images of pristine and oxygen‐plasma treated GaSe flakes. Scale bar is ≈0.6 µm. b) The cross‐sectional TEM image of GaSe with EDS mapping. Scale bar is ≈10 nm.

In addition, to further investigate the oxidation of GaSe, we did AFM, cross‐sectional transmission electron microscopy (TEM) and energy‐dispersive spectroscopy (EDS) of oxidized GaSe thin film. We examined the surface of GaSe before and after oxygen‐plasma by AFM, which clearly demonstrates the existence of oxidation as shown in **Figure** **2**a. The surface roughness of pristine, 4 and 8 s O_2_ plasma treated GaSe thin film is estimated to be 2.5, 10.2, and 14.4 nm respectively from AFM images (Figure S3, Supporting Information). The increase in surface roughness is attributed to oxide phase formation.^[^ ^41^ ^]^ Also, the TEM and EDS elemental mapping images are presented in Figure 2b. Pt layer is deposited on the top of oxidized GaSe thin film to overcome the static charge accumulation during TEM measurement. These images evidently show that a defective oxide layer is formed, having an abrupt confluence with layered GaSe.

**Figure 3:**
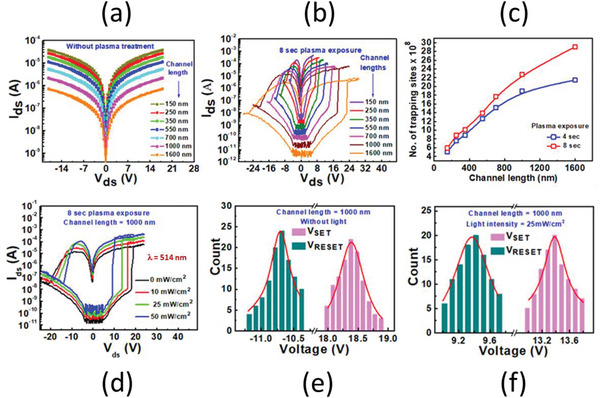
I_ds_–V_ds_ characteristics for the a) pristine Au/GaSe/Au lateral device measured with different channel lengths and for oxygen plasma treatment of b) 8 s, c) Plot of number of trapping sites as a function of channel lengths, d) Light intensity dependent I_ds_–V_ds_ curves of 8 s oxygen plasma treated GaSe device, e) Statistical distribution of V_SET_ and V_RESET_ (e) without light f) and with light intensity of 25 mW cm^−2^ of 8 s oxygen plasma treated GaSe device.

First, we investigated the basic back gate operation of the memtransistor. All the electrical measurements are performed in vacuum condition. Figure S4(a) (Supporting Information) shows the I_ds_‐V_g_ curve of the pristine device. The value of I_ds_ increases on applying the negative V_g_ which clearly shows the p‐type nature of GaSe thin film. Next, we measured the I_ds_‐V_ds_ curves of plasma treated and fresh GaSe devices as shown in **Figure** **3**a,b and Figure S4(b) (Supporting Information). The I_ds_‐V_ds_ characteristics of freshly exfoliated GaSe, and oxygen‐plasma treated devices with different active channel lengths (150, 250, 350, 550, 700, 1000, and 1600 nm). Freshly exfoliated GaSe samples did not demonstrate any resistive switching behavior for all the measured channel lengths as it can be seen in Figure 3a. Subsequently, the bipolar resistive switching was observed in the devices after plasma exposure for 4 and 8 s, see Figure 3b and Figure S3(a) (Supporting Information), respectively. Here, positive biasing of the devices increased the value of the current, which is referred as the low resistance state (LRS) and corresponds to SET process. However, negative sweeping reversed the effect, restoring the device back to a high resistance state (HRS) referring to RESET process. Also, the resistance ratios can be significantly manipulated by varying the channel lengths. In Table S1 (Supporting Information) current ON/OFF ratios, the V_ds_ range and the corresponding lateral electric field intensity for each channel length is mentioned. As it can be seen in the Table S1 (Supporting Information), by increasing the channel length from 150 to 1000 nm, the ON/OFF ratio of current was increased from ≈10^3^ to ≈10^5^ for 4 s plasma treated devices and ≈10^4^ to ≈10^6^ for 8 s plasma treated devices. As the channel length of the device increases, more surface of the channel is exposed to the oxygen plasma, which results in the increase of the off‐state resistance of the device. Further, by increasing the channel length beyond the 1000 nm the results showed the deterioration of current ON/OFF ratio to ≈10^4^ in 4 s and ≈10^5^ in 8 s plasma treated devices. The increase in current ON/OFF ratio of the devices with channel lengths varying from 150 to 1000 nm is explained by considering the effect of oxidation. As more area will be exposed to oxygen plasma, resistance will increase accordingly. In contrast, the decrease in current ON/OFF ratio of the device with channel length 1600 nm as compared to 1000 nm channel length device is attributed to the inverse relation of drain current and channel length. For all channel lengths, the increase in V_SET_ and current ON/OFF ratio (order of 10 magnitude) was observed in 8 s plasma treated GaSe devices as compared to 4 s plasma treated GaSe devices. Therefore, from our results this can be anticipated that plasma exposure is playing the main role in determining the resistive switching behavior of GaSe devices.

To elucidate the role of plasma treatment and to understand the resistive switching phenomena, the I–V curves are fitted with different conduction mechanisms, such as space charge limited current (SCLC), Schottky emission, and Poole Frenkel effect. The I_ds_‐V_ds_ curves of 250 nm channel length with 4 and 8 s plasma exposure time has been chosen for fitting purpose. The best fitting was obtained with the SCLC conduction mechanism, as shown in Figure S5 (Supporting Information). In HRS, two linear regions were identified with the different slopes of 3.3 and 5.8 in 4 s oxygen plasma treated device. Similarly, two linear regions were obtained with slopes of 2.8 and 5 were obtained in 8 s oxygen‐plasma treated device. The SCLC modelling showed that, at a lower value of voltage, most traps are unfilled and, hence, the value of current is small. Tough, at higher voltage, the current increases rapidly due to trap filling. In the present study, the plasma treatment induced defect states is acting as trapping sites for holes. The switching mechanism can be explained by considering the trapping/de‐trapping of charges at defect states. The main purpose of oxygen plasma treatment was to introduce defects (Ga or Se vacancies due to formation of GaO_x_ and SeO_x_) in the GaSe active layer. As it is reported ^[^ ^42^ ^]^ that Ga and Se vacancies create deep defect/trap levels in GaSe band gap. These deep level defects interfere the carriers transport by trapping of carriers in these trap states. As the positive voltage is applied to Au electrode (drain), Fermi level (E_f_) is shifted downward due to the increase in injected holes where the defective states become void of electrons, and holes are captured. As the positive biasing keeps on increasing, more holes are trapped, until all trap levels are filled. After filling all the trap states, additional charges lead to an increase in the current level and the device reaches its LRS. When the negative voltage is applied to Au electrode, the Fermi level is shifted upward, which leads to an emission of holes by defect states and reverting it back to HRS. The current transport in the presence of traps is given by equation 4 ^[^ ^43^, ^44^ ^]^

(4) $$J_{\mathrm{SCLC}}=\;\frac{\mu N_{c}}{q^{m-2}}{\left(\frac{2m-1}{m}\right)}^{m}{\left(\frac{\left(m-1\right)\varepsilon_{o}\varepsilon_{r}}{N_{t}m}\right)}^{m-1}\frac{V^{m}}{L^{2m-1}}$$

where, µ is carrier mobility, N_c_ is the effective density of traps states, N_t_ is the trap density, L is the thickness of the channel, *ε*_o_ is the relative permittivity, *ε*_r_ is the dielectric constant, V is the applied voltage, m can be calculated by the slop of ln(J)‐ ln(V) plot.

The increase in the V_SET_ on increasing the channel length for 4 and 8 s plasma treatment is observed as shown in Figure 3b and Figure S4(b) (Supporting Information). This can be explained by the increase in number of trapping sites. The same interpretation applies for increase in V_SET_ on increasing the plasma treatment time. The increase in plasma treatment time introduces more defects that leads to an increase in V_SET_ of 8 s plasma treated device. The trap density can be calculated by using the formula given by equation 5 ^[^ ^44^ ^]^

(5) $$V_{c}=\;\frac{qN_{t}L^{2}}{2\varepsilon_{r}\varepsilon_{o}}$$

where V_c_ is the critical voltage where all trap states are filled (V_SET_ in this case). As the formula of trapping density is expressed as number of trapping sites per unit volume (specified volume). To calculate volume, which is equal to length × width × height of GaSe flake. The length is calculated from optical image of device, which is equal to 7.7 µm, width is equal to 150 nm, and height is equal to thickness of GaSe flake (42 nm). Then the number of trapping density is calculated as number of trapping sites = trapping density × specified volume. As it can be seen in Figure 3c, by increasing the channel lengths and plasma exposure time, the number of trapping sites are increased that leads to an increase in V_SET_.

To check the stability of the devices, the endurance and retention measurements of the devices with 150, 550, and 1600 nm channel lengths are performed for 4 and 8 s plasma treated devices. The 8 s plasma treated devices showed good endurance up to 500 consecutive cycles, but fluctuation in HRS ad LRS is observed in endurance measurement of 4 s plasma treated devices, see Figure S6 (Supporting Information). Similarly, as shown in Figure S7 (Supporting Information), the retention for 8 s plasma treated devices both LRS and HRS are stable for≈10^4^ s as compared to 4 s plasma devices at room temperature. Though, instability in HRS and LRS is observed for 4 s plasma treated devices. The read voltage (V_ds_) for retention measurement is 2 V. Therefore, our results shows that 4 s plasma treatment is not an optimum time to achieve stable resistive switching which ascribed that in 4 s oxygen‐plasma treated GaSe devices the concentration of defect states is not enough to induce stable resistive switching.

Furthermore, we analyzed the photo assisted resistive switching measurements of oxidized GaSe devices by using laser of wavelength 514 nm with different light intensities where SET and RESET voltages show strong dependence on light intensities. We can see that in Figure 3d and Figure S8(a) (Supporting Information) as the light intensity is increased from 0 to 50 mW cm^−2^, the SET and RESET voltage decreases from 18 to 9 V (for 8 s plasma treated) and from 15 to 7 V (for 4 s plasma treated), respectively in 1000 nm channel length GaSe device. In addition, the variation of V_SET_ at several light intensities on each channel length of GaSe (4 and 8 s plasma treatment devices) is demonstrated in Figure S8 (b) and (c) (Supporting Information). Basically, under the light illumination, a huge amount of electron–hole pairs are produced in the GaSe. These photogenerated holes can be trapped in defect states, so mostly trap states will be filled by the photogenerated holes. So, the number of trap states that need to be filled before achieving the LRS, will be reduced. More holes that are injected from Au electrode can be trapped in the remaining trap states and additional holes lead to increase the current level and device can reach to LRS at a comparatively low voltage. Thus, under the light illumination, the SET voltage of the GaSe device can be lowered, and the big issue of energy consumption of the MEM transistor can be resolved. Moreover, as the intensity of light increases, large amount of photoinduced electron‐hole pairs is generated, that can be trapped in trap states, which further reduces the trap states that need to be filled before achieving the LRS. Likewise, the reduction in the RESET voltage can be assigned to laser‐light assisted de‐trapping of holes from the trap sites. In the presence of laser illumination, charge carriers gain as sufficient energy from laser light, which can easily assist them to de‐trap from trap states relative more conveniently. Such a trend of decrease in SET voltage is observed with different channel lengths in 4 and 8 s oxygen‐plasma treated GaSe devices as shown in Figure S8 (b,c) (Supporting Information). Moreover, the 8 s oxygen‐plasma treated device showed very stable resistive switching with and without light up to ≈10^4^ s as shown in Figure S9 (Supporting Information). These results shows that light exposure on the defected GaSe memtransistor can considerably reduce the power consumption of memtransistor without compromising the stability of the device. The statistical distribution of V_SET_ and V_RESET_ with and without light is presented in Figure 3e,f respectively. This distribution shows that there is a very small variation in the operating voltages of devices even after the light illumination.

**Figure 4:**
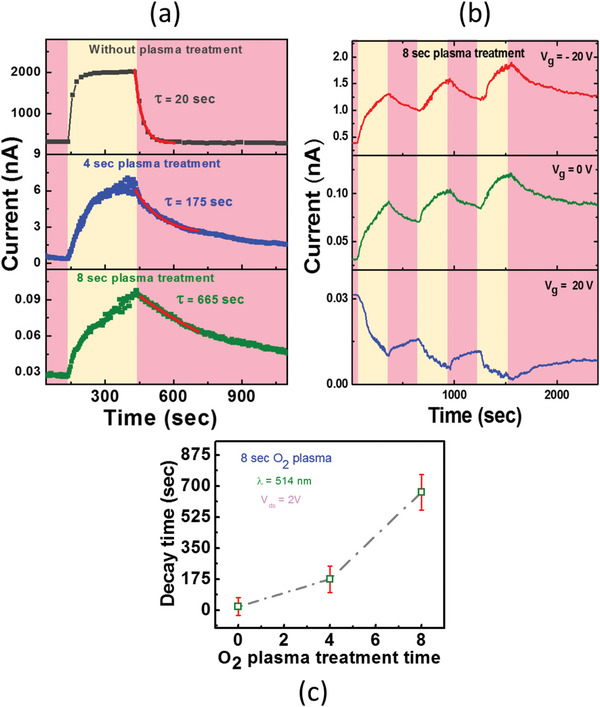
a) Time‐resolved photocurrent measurements of pristine, 4 and 8 s oxygen‐plasma treated GaSe devices at V_g_ = 0 V and V_ds_ = 2 V. b) Gate‐dependent photocurrent measurements of 8 seconds oxygen plasma treated GaSe devices at V_ds_ = 2 V. c) Plot of variation of decay time with O_2_ plasma treatment time.

To further explore the tuning of photo conductance of GaSe devices, we measured the time‐resolved light response of pristine and oxygen‐plasma treated devices as shown in **Figure** **4**a. These Figures presents the photo‐current response of the pristine, 4 and 8 s plasma treated GaSe devices under laser wavelength (*λ* = 514 nm) as a function of time. The V_ds_ is 2 V, and the illumination time is 250 s per cycle for pristine, 4, and 8 s plasma treated GaSe devices. When the device is exposed to laser light, an increase in conductance is observed for pristine, 4 and 8 s plasma treated GaSe devices, respectively. To extract the decay times, photo‐current data is fitted with the following logarithmic equations.

(6) $$I\left(t\right)=I_{o}(e^{-t/t})$$

where I_o_ is the maximum current at a particular time, *τ* is the decay time constant, and t is the response time. Figure 4a shows the exponential fitting of photocurrent data that leads to the extraction of decay time of localized holes in the GaSe defect states. Also, Figure 4b shows the gate dependence of photo response in GaSe memtransistor. At (V_g_ = 0 and ‐20 V) the current increase but at V_g_ = 20 V, the current drops under the laser illumination. In case at V_g_ = 20 V, the NPC appears at positive gate voltage and PPC occurs at negative gate voltage. This implies that the transition between the PPC and NPC is gate tunable in GaSe memory application. Basically, due to oxygen‐plasma treatment, shallow and deep traps are created in GaSe, which are responsible for trapping charge carriers in them. At V_g_ = – 20 V, holes will be injected in GaSe, and the Fermi level will be shifted downward slightly as shown in Figure S10 (a) (Supporting Information). Few injected holes will be trapped in the trap states. Under light exposure, the Fermi level splits into quasi fermi levels (E_fn_ and E_fp_) due to generation of excess carriers (electrons and holes) in the semiconductors as shown in Figure S10 (b) (Supporting Information). As it is p‐type semiconductor, excess holes produced due to light exposure are very small as compared to the concentration of holes in GaSe, So the shift in E_fp_ is very minimal. So, we can assume that E_fp_ = E_f_. The photo‐generated electrons will recombine with trapped holes in trap sates. As a result, excess photo‐generated holes will further increase the conductivity of p‐type GaSe, which results in PPC. However, at Vg = 20 V, electrons will be induced in GaSe, and fermi level will be shifted upward as shown in Figure S10 (c) (Supporting Information). Few electrons will be trapped in the trap states below fermi level. On light exposure, the electrons in trap states will recombine with the photogenerated holes as presented in Figure S10 (d) (Supporting Information), that decrease the conductivity of GaSe. By gate tuning, the transition of PPC to NPC can be utilized to perform a Bolean logic operation in GaSe memtransistor.

Moreover, in Figure 4c, we plotted the decaying time of the GaSe devices as a function of O_2_ plasma exposure time. In pristine GaSe devices, values of decaying time values are ≈2.1 s. After the O_2_ plasma treatment for 4 and 8 s, the temporal response was increased to 175 and 665 s, respectively. This increase in decay time can be contributed to the induced defect/trap states in GaSe during the O_2_ plasma treatment. Since, J. Jiang et al. categorized the decay time in sub milli‐seconds, sub‐seconds, and minutes in term of spatial position of trapping centers within the band gap.^[^ ^45^ ^]^ According to them, decay time in sub milli‐seconds and sub‐seconds belongs to the shallow trap states. The decay constant in minutes corresponds to a combination of shallow and deep traps (mainly deep trap states) within the band gap of a 2D semiconductor. The decay time of the charge carriers in the semiconductor is expressed by

(7) $$\tau \;=\;\tau_{r}+\;\tau_{t}\;\left(1+\;\rho \right)$$

where *τ*_r_ is the recombination time of the excess charge carriers (lifetime of the charge carriers), *τ*_t_ is the time for thermal re‐excitation of trapped charge carriers into the conduction band or valence band. *ρ* is the probability that an electron/hole can be re‐trapped before recombination. The de‐trapping time *τ*_t_, which is inversely proportional to the thermal emission rate of charge carrier's is expressed as

(8) $$\tau_{t}^{-1}=s_{p}N_{v}V_{\mathrm{th}}\exp\left(\frac{-\Delta E}{\mathrm{kT}}\right)$$

where *ρ* denotes the capture cross section of the trap center, N_v_ represents the effective density of states in the valence bands, V_th_ is the thermal velocity of the charge carriers and ΔE is the energy difference between the trap state and the valence band edge (Δ*E* = *E*_t_ − *E*_v_). Therefore, the de‐trapping time could be severely altered when ΔE of trap states varied only by a smaller magnitude. Consequently, a deeper trap state, causing larger ΔE will significantly increase the decay time of the device. Moreover, the number of traps which can respond (de‐trap) to the light exposure significantly depends on the light exposure time. Mostly, those defects having smaller emission lifetimes than the exposure time can respond because holes can't be de‐traped from deep traps in the time scale of seconds. These finding anticipate that relatively long lifetime of the carriers in plasma treated GaSe devices as compared to pristine device is attributed to the presence of deep traps induced by oxygen plasma treatment. As Rak et al. ^[^ ^42^ ^]^ confirmed that Ga and Se vacancies create deep defect levels in GaSe. These results of photo‐response of conductance further confirm that the resistive switching obtained in plasma treated GaSe devices is due to trapping/de‐trapping of holes in trap states.

**Figure 5:**
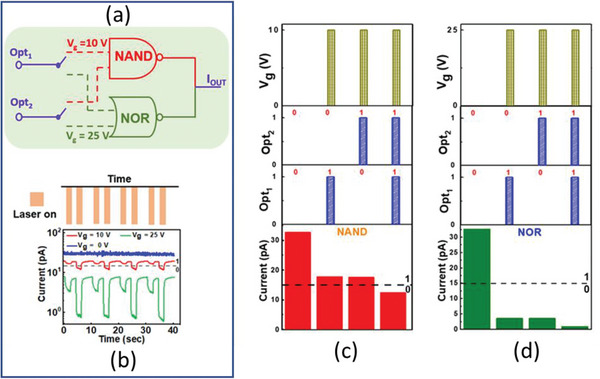
a) Schematic diagram of NAND and NOR logic gates operation, b) Time‐dependent photo‐response of 8 s oxygen‐plasma treated GaSe devices at zero and positive gate voltages. The reprogrammable electrical and optical inputs and outputs of c) NAND and d) NOR logic gates.

Generally, for the operation of Boolean logic devices, two input signals are required to control the output resistance signal of the device. In the present study, for the demonstration of the Boolean logic function in a single device, both electrical and optical signals are utilized to manipulate the resistance state of device. The optical signals are denoted by Opt_1_ (*λ* = 514 nm, intensity = 10 mW cm^−2^, pulse duration = 2 s) and Opt_2_ (laser = 514 nm, intensity = 10 mW cm^−2^, pulse duration 2 s) to control the output signal. The electrical signal “V_g_ = 10 V and V_g_ = 25 V” is used to switch the logical function between “NAND” and “NOR” as schematically presented in **Figure** **5**a.

The hybrid control of electrical and optical inputs on the current level is demonstrated in Figure 5b. The current level below (above) 15 pA is assigned as logical “0” (logical “1”). As it can be seen in Figure 5b neither a single optical input nor only V_g_ = 10 V pulse is sufficient to decrease the current level below 15 pA (logical “0”), until the electrical and both sets of optical pulses are applied. When both V_g_ and optical signals are not applied, logical input values are “0” and “0”. At logical input values of “0” and “0”, the current is at 33 pA, which corresponds to logical “1”. When Vg = 10 V, and only one light signal is applied, logical input values are “0” and “1”. At this point, the current level decrease to ≈17 pA which also corresponds to logical “1”. The logical “0” is obtained only when both V_g_ and two optical signals are applied, which belongs to logical input “1” and “1”. These values of inputs and outputs match with the truth table of the logical NAND gate as shown in Figure S11(a) (Supporting Information). So, by applying Vg = 10 V, and two optical pulses NAND gate operation is achieved as shown in Figure 5c. The truth table of the NOR gate is presented in Figure S11(a) (Supporting Information). According to the truth table of the NOR gate, logical ‘1 is achieved only when both inputs are “0”. As it can be seen in Figures 5b and (d), logical “1” is obtained only when both electrical (V_g_ = 0 V) and optical inputs are “0”. When Vg = 25 V, and one optical signal is applied to the device, current level decrease to ≈3 pA which belongs to logical “0”. The current level decrease further to ≈1 pA (logical “0”), when Vg = 25 V and both optical signals are applied. These values of input and output signals are identical to truth table of NOR gate.

**Figure 6:**
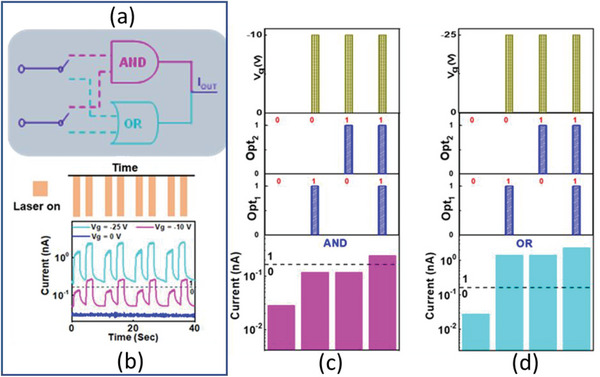
a) Schematic diagram of AND and OR logic gates operation, b) Temporal response of 8 s oxygen‐plasma treated GaSe devices at zero and negative gate voltages. The reprogrammable electrical and optical inputs and outputs of c) AND and d) OR logic gates.

The demonstration of logic function of AND and OR gate is presented in **Figure** **6**. Optical signals denoted by Opt_1_ (laser = 514 nm, intensity = 10 mW cm^−2^, pulse duration = 2 s) and Opt_2_ (laser = 514 nm, intensity = 10 mW cm^−2^, pulse duration = 2 s) are utilized as input signals to control the output signal I_out_. To switch the logical function between “AND” and “OR” the electrical signal “V_g_ = ‐10 and V_g_ = ‐25” is utilized as schematically presented in Figure 6a. The current level below (above) 0.16 nA is assigned as logical “0” (logical “1”) as it can be seen in Figure 6b. It can be seen in Figure S6(b) (Supporting Information), when Vg = ‐10 V and only one light signal is provided current level is below 0.16 nA (logical “0”). The value of current is larger than 0.16 nA (logical “1”) only, when opt_1_ and opt_2_ are simultaneously applied with Vg = ‐10 V (logical inputs “1” and “1”). This corresponds to the truth table of AND gate presented in Figure S11(b) (Supporting Information). For the implementation of the OR operation, the electrical signal (Vg = ‐25 V) is again applied synchronously with optical signals. In this case, the value of current is higher than 0.16 nA, if either opt_1_ and opt_2_ are applied separately or simultaneously with Vg = ‐25 V. Therefore, implementing the logic operation of OR gate as shown in truth table (Figure S11(b), Supporting Information). Therefore, by utilizing these optoelectronic features of the device, the logic functions such as NAND, NOR, AND, and OR can be demonstrated in single device. This feature implies a type of simulation for spatial summation of post synaptic current in biological neural network. In addition, GaSe logic gates shows promising endurance characteristics as shown in Figure S12 (Supporting Information). Figure S13 (Supporting Information) shows the comparison of present device demonstrating four logic gates in single device with the number of devices used for implementing logic gates in previous reports. So, the strategy of creating defect states introduced by oxygen plasma treatment in GaSe layer enabled memory characteristics and multiple logic gates in a single device successfully.

Furthermore, a memtransistor array (1×8) is fabricated and tested to present binary information memory in GaSe memtransistor in the form of American Standard Code for Information Interchange (ASCII) character.

**Figure 7:**
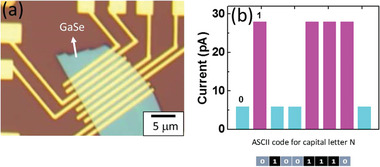
a) Optical microscopic image of 1×8 memristor array device. b) Demonstration of binary coding for capital letter “N”.

Microscopic image of the device is shown in **Figure** **7**a. Eight memory cells are programmed to have the logic states of “0 100 1110” reflecting the uppercase letter “N” as shown in Figure 7b. These findings explored to exploit three‐terminal memtransistors for binary information memory. However, memtransistor of cross bar array design can play multiply‐and‐accumulate operation which possibly executed to unravel data‐base tasks in image detection and AI‐driven applications for voice recognition.

## 3 Conclusions 

In this report, we demonstrated that the defects can be created in the GaSe multilayers by using oxygen‐plasma treatment. This oxygen‐plasma treated GaSe films presented a resistive switching behavior with current ON/OFF ratio of ≈10^4^ to ≈10^6^ depending on the channel lengths (150, 250, 350, 550, 700, 1000, and 1600 nm). The underlying mechanism for resistive switching behavior is assigned to trapping/de‐trapping of charge carriers in trap states introduced by oxygen‐plasma treatment. The numbers of trap states are encouraged by increasing the exposure time of oxygen‐plasma treatment from 4 to 8 s. Also, the current ON/OFF ratio of the devices show a strong dependence on the plasma treatment time. This dependence is explained by the formation of oxide layer by prolong the exposure time. Further, we explore the laser‐assisted behavior of resistive switching to reveal the trapping/de‐trapping mechanism under light. In addition, we also investigated the gate dependent (Vg = ‐20, 0, and 20 V) transition of PPC to NPC in GaSe mem‐transistors. By utilizing this feature of devices, four logic gates OR, AND, NOR, and NAND are demonstrated in a single device. Furthermore, a memtransistor array (1×8) of GaSe is programmed to have logic states of “0 100 1110” reflecting the uppercase letter “N”. We believe that our study will provide the new avenue for cutting‐edge technology in neuromorphic and multifunctional electronic applications.

## 4 Experimental Details

The p‐type Si substrate with thermally grown SiO_2_ layer of 300 nm was utilized as a back gate where multi‐layer GaSe flakes were exfoliated by scotch tape and directly transferred on top of SiO_2_ by a polydimethylsiloxane (PDMS) stamp. Subsequently, the electrodes were patterned by electron beam lithography followed by Cr/Au (6/40 nm) deposition by the thermal evaporation. Afterward, the top surface layers of GaSe surface were treated with mild oxygen plasma by reactive ion etching tool. The RF power and gas flow rate during oxygen plasma treatment were 50 W and 10 sccm, with the treatment times of 4 and 8 s.

The surface composition was investigated by a Thermo Fisher Scientific (with K‐alpha X‐ray source) X‐ray photoelectron Spectroscopy (XPS). To confirm the oxide formation on the surface of GaSe, a Renishaw micro‐spectrometer with a laser wavelength of 514 nm was used to obtain Raman spectra. The thickness of the GaSe flakes was measured by atomic force microscopy (AFM). The VEECO Dimension 3100 AFM was utilized for thickness measurements. The HITACHI S‐4700 field effect scanning electron microscopy (FE‐SEM) was used to capture the image of the device layout. The electrical measurements were performed by using a Keithley 2400 (Beaverton, OR, USA) and pico‐ammeter Keithley 6485 (Beaverton, OR, USA).

## Conflict of Interest

The authors declare no conflict of interest.

## Supporting information

Supporting Information

## Data Availability

The data that support the findings of this study are available from the corresponding author upon reasonable request.
