# Supplementary material for: Optically Reconfigurable Complementary Logic Gates Enabled by Bipolar Photoresponse in Gallium Selenide Memtransistor
Source: Adv Sci (Weinh). 2023 Apr 19;10(17):2205383. doi: 10.1002/advs.202205383 (PMC10265048; doi:10.1002/advs.202205383)
Supplement: Supplementary file 1 — Supporting Information [file ADVS-10-2205383-s001.pdf]

ADVANCED SCIENCE
Open Access

Supporting Information

for *Adv. Sci.*, DOI 10.1002/advs.202205383

Optically Reconfigurable Complementary Logic Gates Enabled by Bipolar Photoresponse in Gallium Selenide Memtransistor

Shania Rehman, Muhammad Asghar Khan, Honggyun Kim, Harshada Patil, Jamal Aziz, Kalyani D. Kadam, Malik Abdul Rehman, Muhammad Rabeel, Aize Hao, Karim Khan, Sungho Kim, Jonghwa Eom, Deok-kee Kim and Muhammad Farooq Khan**

Supplementary Information

Optically Reconfigurable Complementary Logic Gates Enabled by Bipolar Photoresponse of Gallium Selenide Memtransistor

Shania Rehman[1#], Muhammad Asghar Khan[2#], Hongyun Kim[1#], Harshada Patil[3], Jamal Aziz[3], Kalyani D. Kadam[3], Malik Abdul Rehman[4], Muhammad Rabeel[3], Aize Hao[5], Karim Khan[6], Sungho Kim[1], Jonghwa Eom[2], Deok-kee Kim*[1,3] and Muhammad Farooq Khan[3]*

[1]Department of Semiconductor System Engineering, Sejong University, 209 Neungdong-ro, Gwangjin-gu, Seoul 05006, Korea.

[2]Department of Physics & Astronomy and Graphene Research Institute, Sejong University, Seoul 05006, Republic of Korea.

[3]Department of Electrical Engineering, Sejong University, 209 Neungdong-ro, Gwangjin-gu, Seoul 05006, Korea.

[4] Department of Chemical Engineering, New Uzbekistan University, Tashkent, Uzbekistan.

[4]State Key Laboratory of Chemistry and Utilization of Carbon-Based Energy Resources, College of Chemistry, Xinjiang University, Urumqi, 830017, Xinjiang, PR China.

[6]School of Mechanical Engineering, Dongguan University of Technology, Dongguan, 523808, China.

# These authors contributed equally

*Corresponding authors

E-mail addresses: mfk@sejong.ac.kr and deokkeekim@sejong.ac.kr

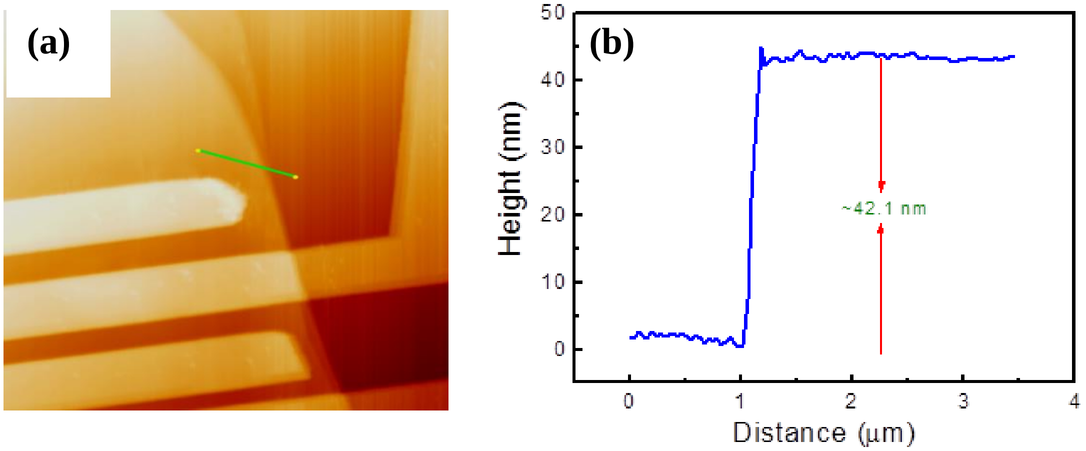

Figure S1. **(a)** AFM image of multilayered GaSe, and **(b)** the corresponding height profile of the GaSe flake along the green line shown in the AFM image.

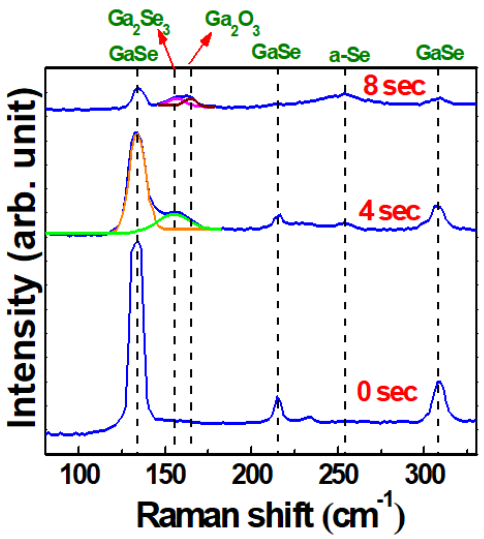

Figure S2. Raman spectra of pristine, 4- and 8-seconds oxygen plasma treated GaSe samples.

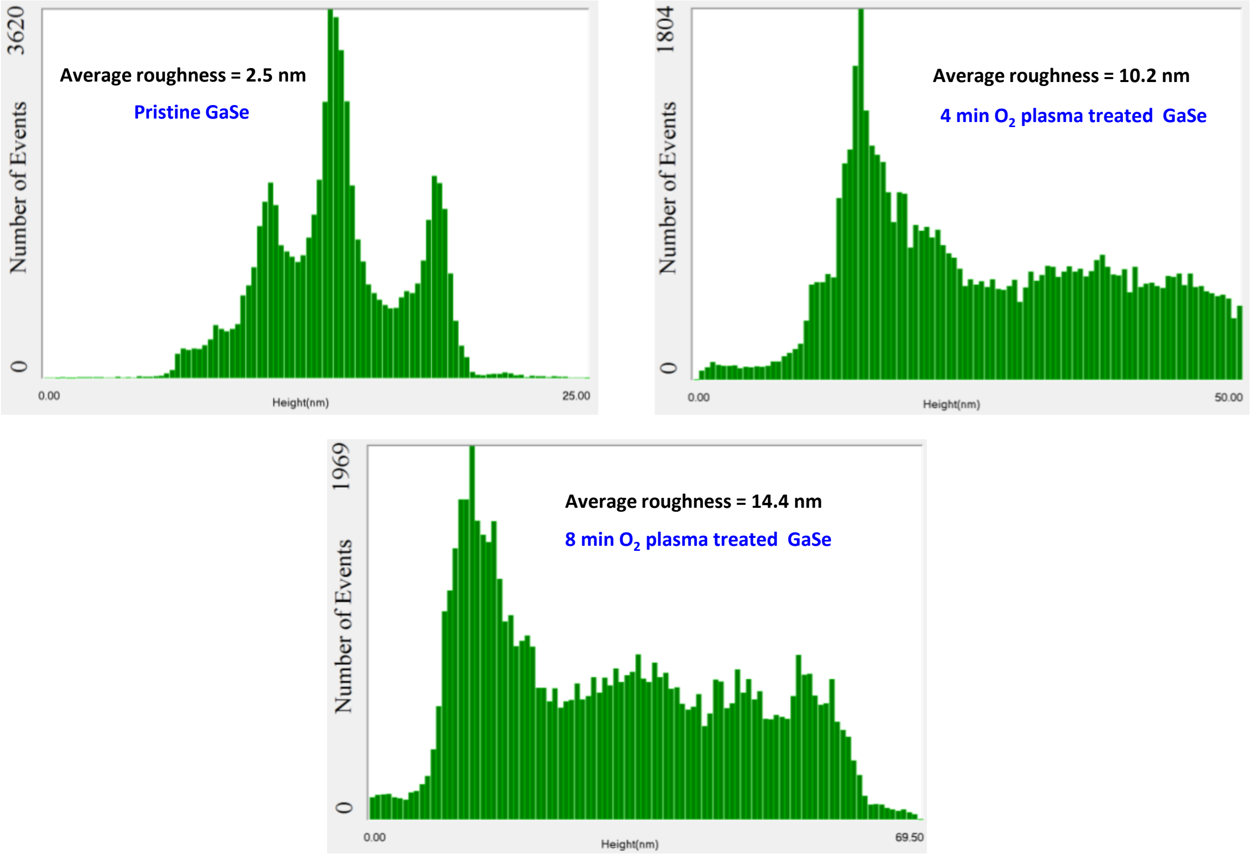

Figure S3: The average roughness of pristine, 4, and 8 seconds O_2 plasma treated GaSe thin films.

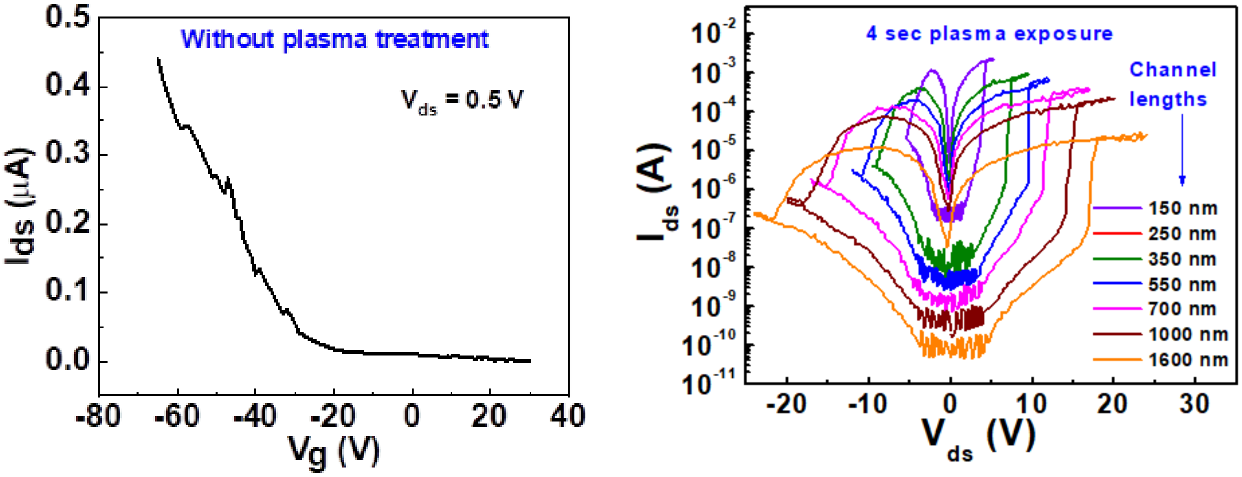

(a) **(b)**

Figure S4. (a) I_{ds}-V_g characteristics for the pristine GaSe device with 1000 nm channel length, where V_{ds} is fixed at 0.5 V **(b)** I_{ds}-V_{ds} characteristics for the GaSe device measured with different channel lengths for 4- second oxygen plasma treatment time.

Table S1

O_2 Plasma treatment time (seconds)	Channel length (nm)	$I_{On/Off}$	V_{ds} range (V)	Electric field intensity (MV/cm)
4	150	3.4×10^{3}	0 to ±5	0.33
	250	6.1×10^{3}	0 to ±8	0.32
	350	1.7×10^{4}	0 to ±9.5	0.27
	550	4.2×10^{4}	0 to ±12	0.22
	700	6.7×10^{4}	0 to ±17	0.24
	1000	2.5×10^{5}	0 to ±20	0.20
	1600	7.5×10^{4}	0 to ±24	0.15
8	150	4.2×10^{4}	0 to ±6	0.4
	250	6.7×10^{4}	0 to ±9	0.36
	350	9.1×10^{4}	0 to ±11	0.32
	550	1.9×10^{5}	0 to ±13	0.23
	700	4.4×10^{5}	0 to ±17	0.24
	1000	2.7×10^{6}	0 to ±24	0.24
	1600	3.6×10^{5}	0 to ±29	0.18

Table S1: The comparison of I_{on}/I_{off} ratio, V_{ds} range and corresponding electric fields at different channel lengths for 4- and 8-seconds oxygen plasma treated GaSe devices.

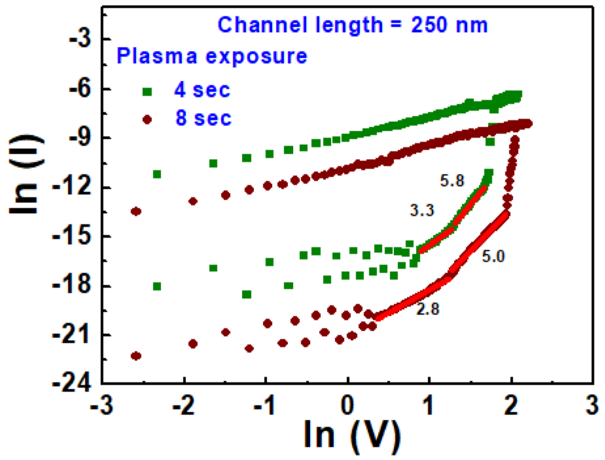

Figure S5: Fitting of SCLC conduction mode for 4, and 8 sec O_2 plasma treated GaSe devices with 250 nm channel length.

(a)

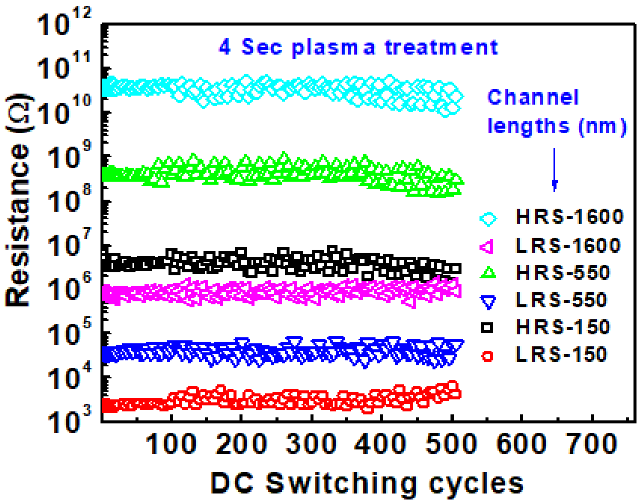

(b)

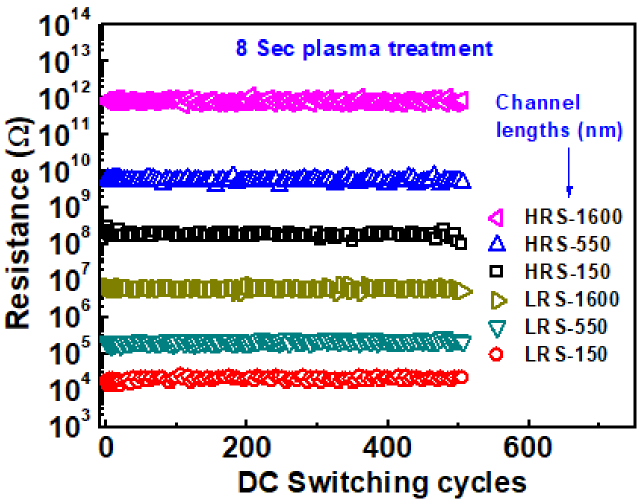

Figure S6. Endurance measurement of Au/GaSe/Au device with different channel lengths for **(a)** 4-seconds and **(b)** 8-seconds plasma treatment time.

(a)

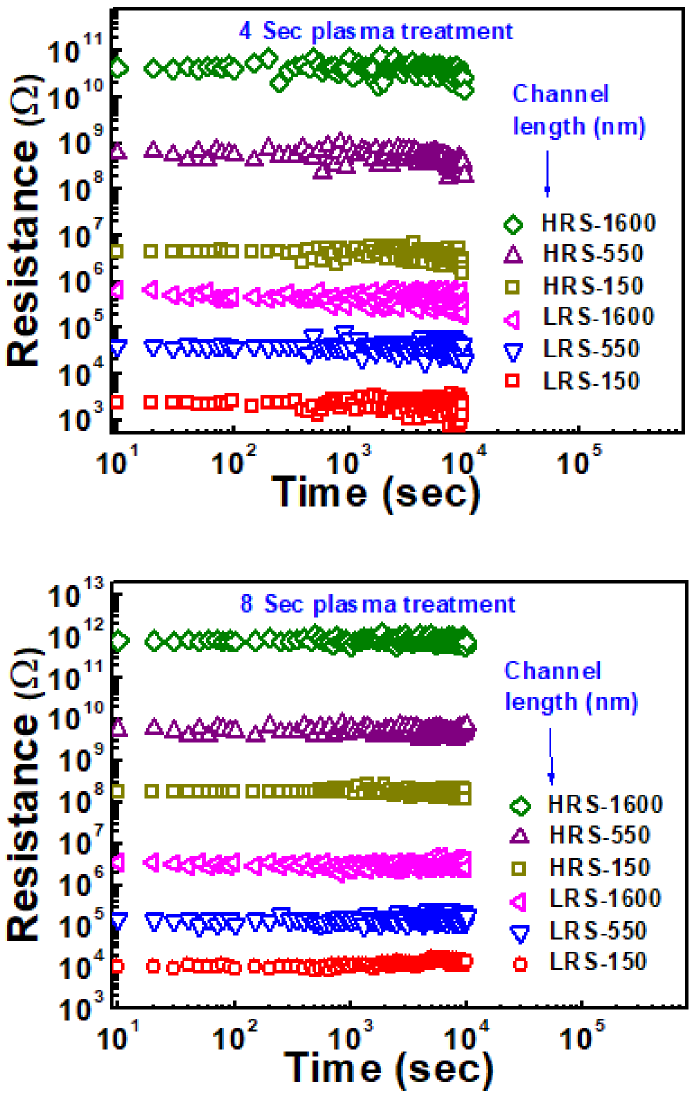

(b)

Figure S7. Retention measurement of Au/GaSe/Au lateral device with different channel lengths for **(a)** 4-seconds and **(b)** 8-seconds plasma treatment time.

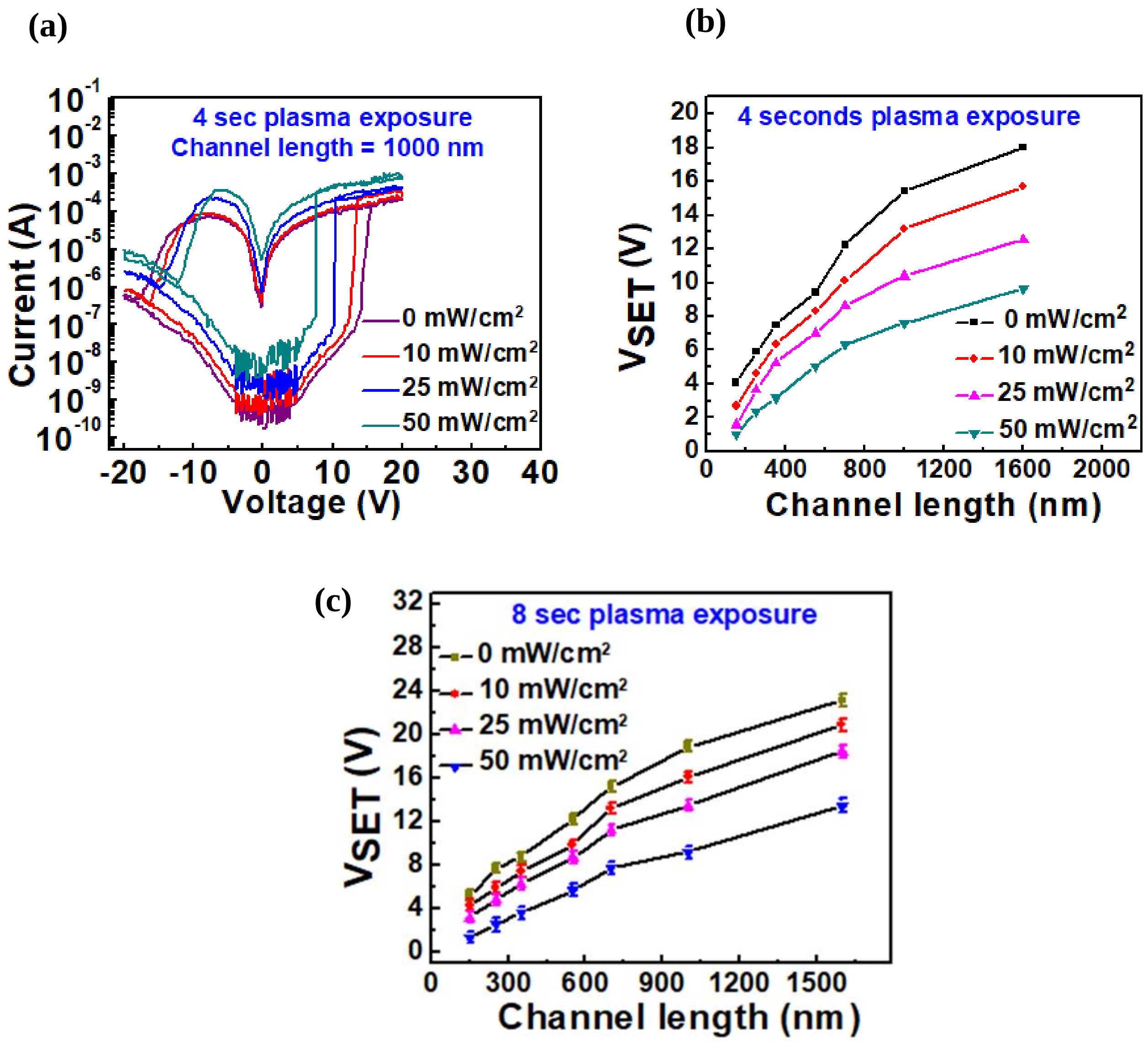

Figure S8. (a) Light intensity dependent I_{ds}-V_{ds} curves, and **(b)** Variation of V_{SET} with respect to different channel length at different light intensities, for 4-seconds oxygen plasma treated GaSe device. **(c)** Variation of V_{SET} with respect to different channel length at different light intensities, for 8-seconds oxygen plasma treated GaSe device.

(a)

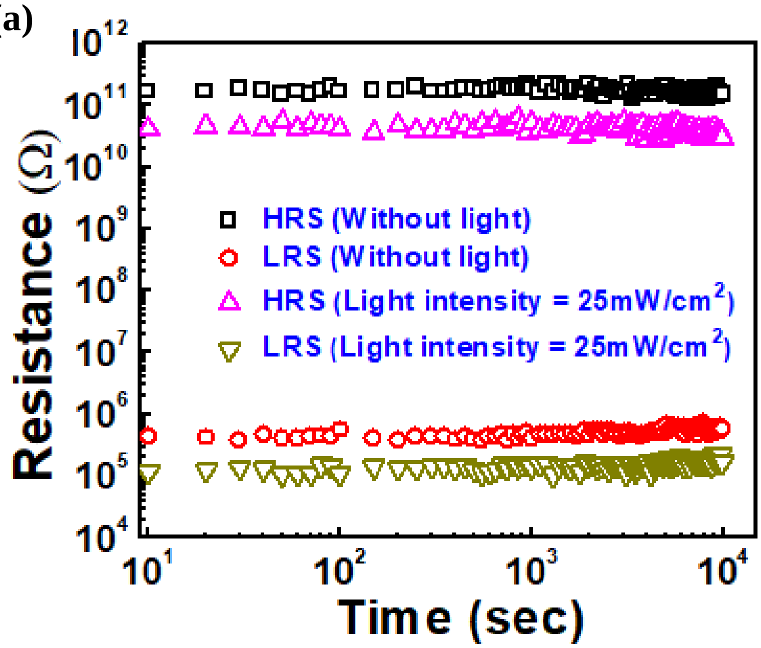

Figure S9. Comparison of Retention measurement of Au/GaSe/Au device (channel length 1000 nm) without light and with light intensity of 25mW/cm^2.

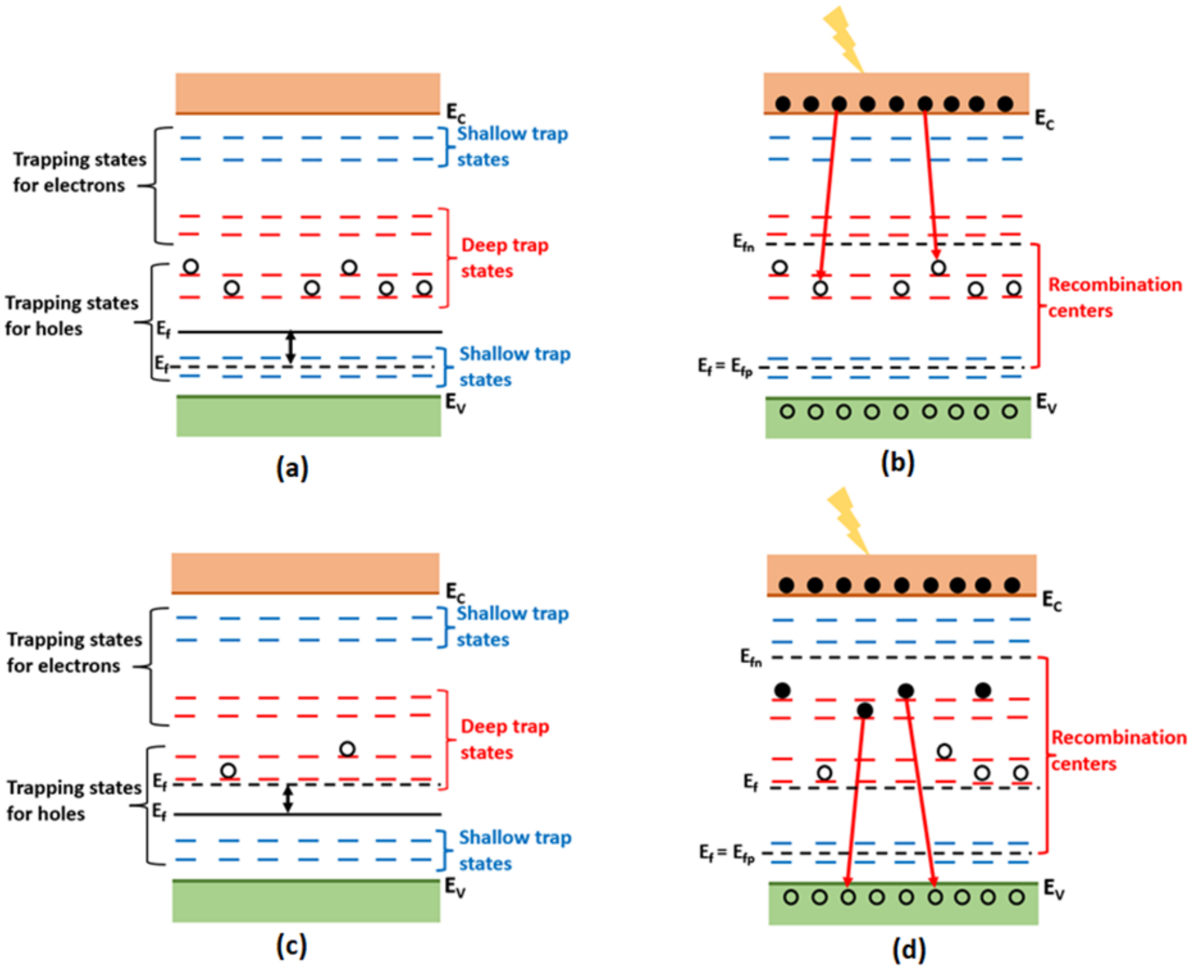

Figure S10. Energy band diagram of Au/GaSe/Au device **(a)** Negative gate. **(b)** Negative gate voltages under illumination. **(c)** Positive gate. **(d)** Positive gate under UV illumination.

(a)

		NAND	NOR
Input		Output	
Opt_1	Opt_2	I_{OUT}	
0	0	1	1
1	0	1	0
0	1	1	0
1	1	0	0

(b)

		AND	OR
Input		Output	
Opt_1	Opt_2	I_{OUT}	
0	0	0	0
1	0	0	1
0	1	0	1
1	1	1	1

Figure S11. Truth table of logic **(a)** NAND and **(b)** NOR gates.

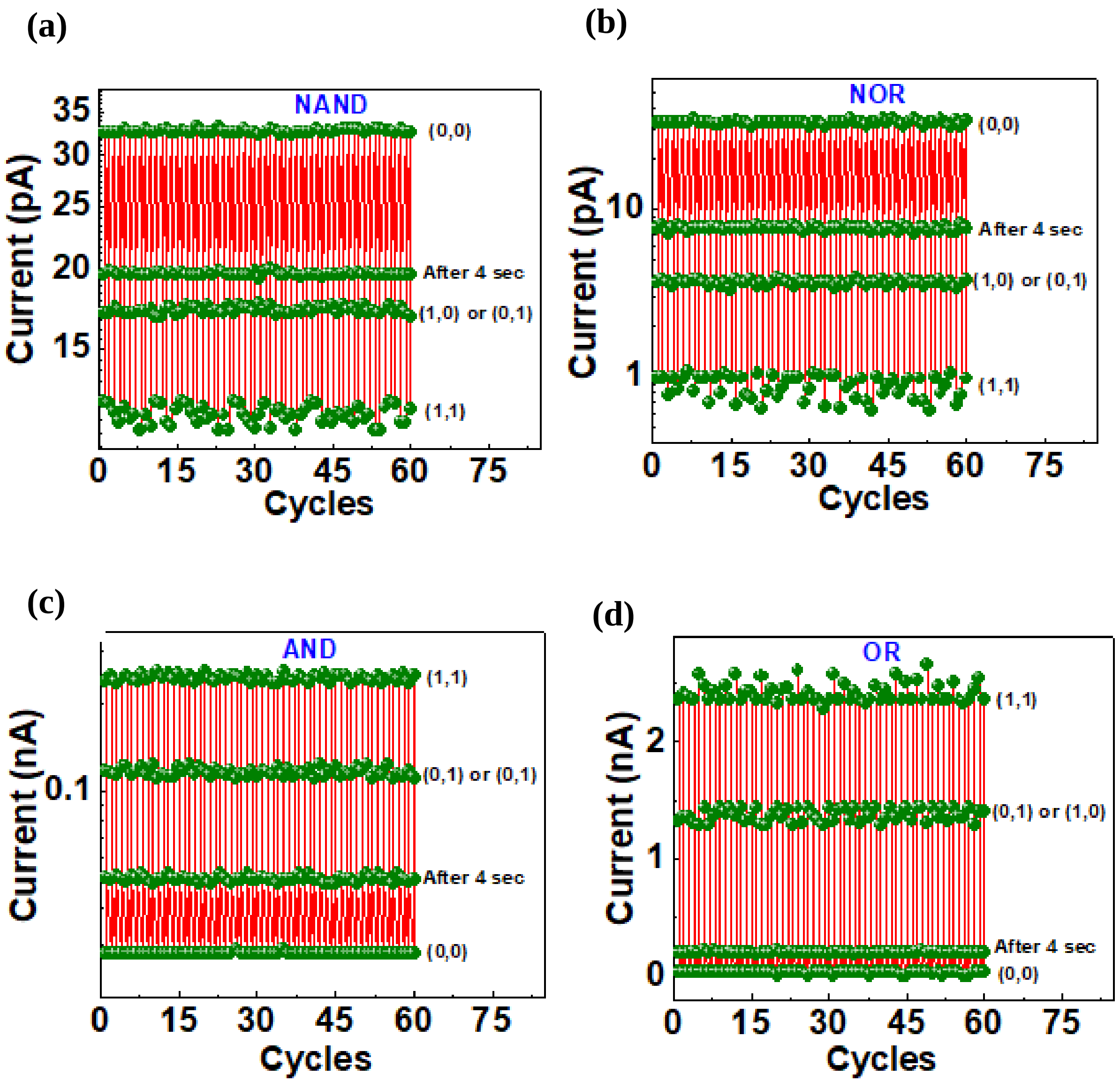

Figure S12. Cyclic reconfiguration of **(a)** "NAND", **(b)** "NOR", **(c)** "AND" and **(d)** "OR" gate showing the switching between logical '0' and '1' state and finally back to initial state after 4 seconds for 60 cycles.

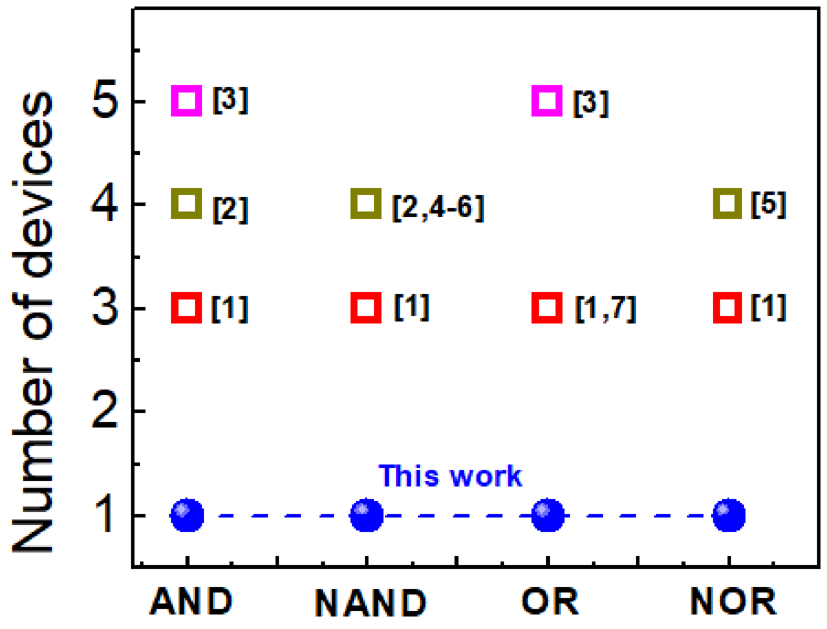

Figure S13. Comparison of number of devices employed in present work for logic gates operation with the previously reported work [1-7].

.

References:

1. Luo, L., et al., *Memristor-based stateful logic gates for multi-functional logic circuit.* 2020. **14**(6): p. 811-818.
2. Huang, P., et al., *Reconfigurable nonvolatile logic operations in resistance switching crossbar array for large-scale circuits.* 2016. **28**(44): p. 9758-9764.
3. Krishnaprasad, A., et al., *MoS2 synapses with ultra-low variability and their implementation in Boolean logic.* 2022. **16**(2): p. 2866-2876.
4. Balatti, S., S. Ambrogio, and D.J.I.t.o.E.D. Ielmini, *Normally-off logic based on resistive switches—Part I: Logic gates.* 2015. **62**(6): p. 1831-1838.
5. Sun, Z., et al., *Logic computing with stateful neural networks of resistive switches.* 2018. **30**(38): p. 1802554.
6. He, Z.-Y., et al., *Atomic layer-deposited HfAlOx-based RRAM with low operating voltage for computing in-memory applications.* 2019. **14**(1): p. 1-5.
7. Liu, Q., et al., *Multifunctional Optoelectronic Random Access Memory Device Based on Surface-Plasma-Treated Inorganic Halide Perovskite.* 2021. **7**(7): p. 2100366.
